# Supplementary material for: A fault-intrusion-tolerant system and deadline-aware algorithm for scheduling scientific workflow in the cloud
Source: PeerJ Comput Sci. 2021 Nov 2;7:e747. doi: 10.7717/peerj-cs.747 (PMC8576554; doi:10.7717/peerj-cs.747)
Supplement: Supplemental Information 6 [file peerj-cs-07-747-s006.zip › Myproject1.1/docs/org/cloudbus/cloudsim/power/models/package-tree.html]

org.cloudbus.cloudsim.power.models Class Hierarchy


JavaScript is disabled on your browser.


- Overview
- Package
- Class
- Use
- Tree
- Deprecated
- Index
- Help

- Prev
- Next

- Frames
- No Frames

- All Classes

# Hierarchy For Package org.cloudbus.cloudsim.power.models

Package Hierarchies:

- All Packages

## Class Hierarchy

- java.lang.Object
  - org.cloudbus.cloudsim.power.models.PowerModelCubic (implements org.cloudbus.cloudsim.power.models.PowerModel)
  - org.cloudbus.cloudsim.power.models.PowerModelLinear (implements org.cloudbus.cloudsim.power.models.PowerModel)
  - org.cloudbus.cloudsim.power.models.PowerModelSpecPower (implements org.cloudbus.cloudsim.power.models.PowerModel)
    - org.cloudbus.cloudsim.power.models.PowerModelSpecPowerHpProLiantMl110G3PentiumD930
    - org.cloudbus.cloudsim.power.models.PowerModelSpecPowerHpProLiantMl110G4Xeon3040
    - org.cloudbus.cloudsim.power.models.PowerModelSpecPowerHpProLiantMl110G5Xeon3075
    - org.cloudbus.cloudsim.power.models.PowerModelSpecPowerIbmX3250XeonX3470
    - org.cloudbus.cloudsim.power.models.PowerModelSpecPowerIbmX3250XeonX3480
    - org.cloudbus.cloudsim.power.models.PowerModelSpecPowerIbmX3550XeonX5670
    - org.cloudbus.cloudsim.power.models.PowerModelSpecPowerIbmX3550XeonX5675
  - org.cloudbus.cloudsim.power.models.PowerModelSqrt (implements org.cloudbus.cloudsim.power.models.PowerModel)
  - org.cloudbus.cloudsim.power.models.PowerModelSquare (implements org.cloudbus.cloudsim.power.models.PowerModel)

## Interface Hierarchy

- org.cloudbus.cloudsim.power.models.PowerModel

- Overview
- Package
- Class
- Use
- Tree
- Deprecated
- Index
- Help

- Prev
- Next

- Frames
- No Frames

- All Classes
